# Gender dysphoria: prejudice from childhood to adulthood, but no impact on inflammation. A cross-sectional controlled study

**DOI:** 10.47626/2237-6089-2020-0007

**Published:** 2021-02-26

**Authors:** André Gonzales Real, Anna Martha Vaitses Fontanari, Angelo Brandelli Costa, Bianca Machado Borba Soll, Giovana Bristot, Larissa Fagundes de Oliveira, Ana Maria Kamphorst, Maiko Abel Schneider, Maria Inês Rodrigues Lobato

**Affiliations:** 1 Programa de Identidade de Gênero Hospital de Clínicas de Porto Alegre Universidade Federal do Rio Grande do Sul Porto AlegreRS Brazil Programa de Identidade de Gênero (PROTIG), Hospital de Clínicas de Porto Alegre (HCPA), Universidade Federal do Rio Grande do Sul (UFRGS), Porto Alegre, RS, Brazil.; 2 Programa de Pós-Graduação em Psiquiatria e Ciências do Comportamento UFRGS Porto AlegreRS Brazil Programa de Pós-Graduação em Psiquiatria e Ciências do Comportamento, UFRGS, Porto Alegre, RS, Brazil.; 3 Departamento de Psicologia Pontifícia Universidade Católica do Rio Grande do Sul Porto AlegreRS Brazil Departamento de Psicologia, Pontifícia Universidade Católica do Rio Grande do Sul (PUCRS), Porto Alegre, RS, Brazil.; 4 INCT Translacional em Medicina Hospital de Clinicas de Porto Alegre UFRGS Porto AlegreRS Brazil Laboratório de Psiquiatria Molecular, INCT Translacional em Medicina, Hospital de Clinicas de Porto Alegre (HCPA), UFRGS, Porto Alegre, RS, Brazil.; 5 Programa de Pós-Graduação em Bioquímica UFRGS Porto AlegreRS Brazil Programa de Pós-Graduação em Bioquímica, UFRGS, Porto Alegre, RS, Brazil.

**Keywords:** Gender dysphoria, childhood maltreatment, discrimination, inflammatory cytokines, transsexuality, case-control study

## Abstract

**Introduction:**

Gender dysphoria (GD) is characterized by a marked incongruence between experienced gender and one’s gender assigned at birth. Transsexual individuals present a higher prevalence of psychiatric disorders when compared to non-transsexual populations, and it has been proposed that minority stress, i.e., discrimination or prejudice, has a relevant impact on these outcomes. Transsexuals also show increased chances of having experienced maltreatment during childhood. Interleukin (IL)-1β, IL-6, IL-10 and tumor necrosis factor-alpha (TNF-α) are inflammatory cytokines that regulate our immune system. Imbalanced levels in such cytokines are linked to history of childhood maltreatment and psychiatric disorders. We compared differences in IL-1β, IL-6, IL-10 and TNF-α levels and exposure to traumatic events in childhood and adulthood in individuals with and without GD (DSM-5).

**Methods:**

Cross-sectional controlled study comparing 34 transsexual women and 31 non-transsexual men. They underwent a thorough structured interview, assessing sociodemographic information, mood and anxiety symptoms, childhood maltreatment, explicit discrimination and suicidal ideation. Inflammatory cytokine levels (IL-1β, IL-6, IL-10 and TNF-α) were measured by multiplex immunoassay.

**Results:**

Individuals with GD experienced more discrimination (p = 0.002) and childhood maltreatment (p = 0.046) than non-transsexual men. Higher suicidal ideation (p < 0.001) and previous suicide attempt (p = 0.001) rates were observed in transsexual women. However, no differences were observed in the levels of any cytokine.

**Conclusions:**

These results suggest that transsexual women are more exposed to stressful events from childhood to adulthood than non-transsexual men and that GD *per se* does not play a role in inflammatory markers.

## Introduction

According to the Diagnostic and Statistical Manual of Mental Disorders, 5th edition (DSM-5), gender dysphoria (GD) is characterized by a marked incongruence between experienced gender and one’s gender assigned at birth.^1^ GD is a rare condition: the estimated prevalence varies from 1:20,000 to 1:10,000 for transsexual women.^1^ The only existing metanalysis about prevalence of GD found a rate of 6.8/100,000 for transsexual women.^2^

Transsexual persons are exposed to traumatic experiences during their lifetime: from childhood to adulthood. Accordingly, gender-variant children are at high risk of exposure to maltreatment.^3^ Sexual minorities, including transsexuals, have increased chances of having been abused during childhood.^4 , 5^ Childhood maltreatment has been considered a risk factor for different psychopathologies in adult life, such as mood and anxiety disorders, post-traumatic stress disorder, suicidal ideation, self-harm behavior and antisocial personality disorder.^6 - 8^ This kind of emotional turmoil can also be seen during the bereavement process of a loss of a significant other by suicide, which in turn can increase suicide risk in the survivors.^9^ In addition, children exposed to maltreatment can suffer from poor social skills and emotional dysregulation, which can influence worse outcomes in mental health.^8^ During their youth, transsexual women are commonly exposed to a stressful environment and engage in behaviors that place them at risk for sexually transmitted infections.^10 , 11^ Finally, transsexual adults are victimized by high prevalences of HIV and difficulties accessing health care.^12 , 13^

Meyer has proposed the minority stress theory to better explain the impact of prejudice in sexual minorities.^14 , 15^ This model is based on the societal reaction theory, meaning that specific social conditions act as stressors, which, if not moderated by adequate coping resources, can lead to disorders.^15^ The distress which sexual minorities are subject to include three factors: a) direct experience of prejudice, through violence or difficult access to public policies, for example; b) expectation of discrimination, i.e., the perception that one’s sexual orientation or gender identity will not be accepted; c) internalized prejudice, which refers to negative beliefs that the person has about their own sexual orientation and gender identity.^14^ Although the minority stress theory was initially based on gay men, it has been applied to transsexual people.^16^ Minority stress theory has helped to better explain mental health disparities found between transsexual and non-transsexual persons: transsexual individuals present a higher prevalence of psychiatric disorders when compared to non-transsexual populations, especially suicidal behavior, mood and anxiety disorders.^17 - 25^

Cytokines are relatively large proteins that act as messengers regulating our immune system.^26^ They are secreted mostly by white cells (mainly T lymphocytes, macrophages and monocytes) in the periphery, while in the brain they are secreted by astrocytes and microglia.^26 - 28^ Examples of pro-inflammatory cytokines include interleukin (IL)-1β, IL-6 and tumor necrosis factor-alpha (TNF-α); IL-10, in turn, is an anti-inflammatory cytokine. One theory for the development of psychopathologies, such as mood and anxiety disorders, is the cytokine hypothesis, which proposes that psychopathology can be caused by an imbalance in cytokine levels.^29^ Several studies have confirmed the cytokine hypothesis. For example, both chronic and acute exposure to cytokines mimic depressive symptoms.^30 , 31^ Furthermore, several studies have shown altered levels of inflammatory cytokines (particularly IL-1β, IL-6, TNF-α and IL-10) in many different psychopathologies, such as major depressive disorder, bipolar disorder, obsessive-compulsive disorder, anxiety disorders (more precisely panic disorder and generalized anxiety disorder), schizophrenia and post-traumatic stress disorder.^26 , 32 - 41^ In addition, experiencing traumatic events during both childhood and adulthood has been associated with alterations in inflammatory cytokines.^42 - 44^

There are few studies assessing biomarkers in GD, and those focus exclusively on brain-derived neurotrophic factor (BDNF). BDNF is the neurotrophin most abundantly expressed in the central nervous system.^45^ BDNF is associated with synaptic plasticity, neurogenesis, neuronal survival and maturation of neuronal pathways.^46 , 47^ Traumatic events^48 , 49^ as well as psychiatric disorders,^50^ such as bipolar mood disorder,^49 , 51 , 52^ Major depressive disorder^53^ and schizophrenia,^54^ are associated with lower levels of BDNF. In a previous study, our group observed that serum levels of BDNF were significantly lower (p = 0.003) in transsexual women when compared to non-transsexual men.^55^ We suggested that BDNF acted as a biomarker to chronic exposure to minority stressors.^13^ In response, Fuss et al. assessed, through a longitudinal study, the impact of hormone therapy on BDNF levels in 20 transsexual women. After 12 months of hormone therapy, a reduction of BDNF serum concentration was found (p = 0.014).^56 , 57^ In contrast, Auer et al., using the same methodology, did not find differences (p = 0.795) in BDNF levels before and after hormone therapy in 29 transsexual men.^58^ Therefore, it was concluded that testosterone therapy for transsexual men does not influence BDNF serum levels. In another study, searching for similar data for transsexual men, we found a reduction of BDNF serum levels in the transsexual population when compared to non-transsexual women (p = 0.027) and non-transsexual men (p = 0.035).^59^

In this regard, people diagnosed with GD are chronically exposed to stigma and prejudice, often reflected by exposure to traumatic events like physical and sexual violence,^60^ and they experience a high prevalence of childhood maltreatment.^61^ There is evidence that being under such stressors may contribute to the onset of psychiatric comorbidities in the future. In addition, it has been considered that imbalanced inflammatory cytokine levels are linked to childhood maltreatment and that this imbalance plays a role in the development of mental disorders. We hypothesized that experiencing stressful situations, like discrimination and childhood maltreatment, could lead to an imbalance in inflammatory cytokines and, perhaps, psychiatric disorders in GD. In this study, we aim to evaluate differences in IL-1β, IL-6, IL-10 and TNF-α levels and exposure to traumatic events in childhood and adulthood between individuals with and without GD.

## Methods

### Participants

This cross-sectional controlled study was conducted at the outpatient clinic of the Programa de Identidade de Gênero (PROTIG) from Hospital de Clínicas de Porto Alegre (HCPA). Thirty-four transsexual women were recruited from the PROTIG clinic and had to meet DSM-5 criteria for GD.^1^ None of them had undergone gender affirmation surgery when the assessment was taken. Thirty-four non-transsexual men were recruited while waiting for their relatives in any other outpatient clinic at HCPA and among men who were volunteering to donate blood at the blood bank of the same hospital. This sample size is compatible with other studies that evaluated inflammatory cytokines in case-controlled studies, which recruited similar (n = 73),^34^ or smaller samples (n = 40,^62^ n = 45,^63^ n = 36,^64^ n = 53,^65^ n = 50^66^). Exclusion criteria for both groups were: being under 18 years of age; having a diagnosis of characteristically inflammatory systemic diseases, particularly HIV, due to its possible effects on inflammatory cytokines^67^; and having a diagnosis of mental retardation, dementia, substance use disorder or psychotic disorders, which were assessed by The Mini International Neuropsychiatric Interview 6.0 (MINI 6.0).^68 , 69^ Participants on psychotropic or anti-inflammatory medications were excluded, due to the influence of these drugs on the analyzed biomarkers.^62 , 70 - 72^ Three transsexual women were excluded because they were diagnosed with HIV when control blood tests were analyzed.

### Measures

Both groups underwent a structured interview protocol built for this study, where sociodemographic information, mood and anxiety symptoms, childhood maltreatment, explicit discrimination and suicidal ideation were evaluated. Mood and anxiety symptoms were assessed using the Depression, Anxiety and Stress Scale (DASS-21).^73 , 74^ This scale has 21 questions divided into three subscales that assess depression, anxiety and stress; a score was calculated for each subscale. Childhood maltreatment was assessed using the 28-item Childhood Trauma Questionnaire (CTQ).^75 , 76^ This instrument assesses, in five subscales, childhood emotional, physical and sexual abuse, and physical and emotional neglect; scores were counted for each subscale. Explicit discrimination was assessed using the Brazilian Explicit Discrimination Scale (BEDS),^77 , 78^ which measures general experiences of prejudice and stigma in an 18-item questionnaire (examples of experiences include bullying or violence from police, family members or unknown individuals). The Columbia Suicide Severity Rating Scale (C-SSRS) assessed suicidal ideation and behavior.^79^ All scales were validated to Portuguese. After the interview, the participants were invited to have blood samples drawn for biochemical analysis.

### Biochemical assays

Blood samples were collected by venipuncture from each patient or control and allowed to clot in blood collection tubes with no additive. Subsequently, the whole blood was centrifuged for 10 minutes at 1,000 xg and the serum was removed, aliquoted and stored at -80 ºC until assayed. Blood and platelet counts and anti-HIV antibodies were also measured to control for possible confounders.

### Cytokine levels

IL-1β, IL-6, IL-10 and TNF-α serum levels were measured by multiplex immunoassay using the commercial kit ProcartaPlex™ Multiplex Immunoassay - Human Custom HS ProcartaPlex 4-plex (PPXS-04-MXU626J; Invitrogen, Austria), according to the manufacturer’s instructions. In brief, magnetic beads were pipetted into all wells, the plate was washed, and after the addition of assay buffer, each diluted standard and samples (without dilution) were added into the appropriate wells and the plate was sealed and incubated on the plate shaker overnight at 4 ºC. Once completed, the plate was washed two times followed by the addition of detection antibodies into each well and, after 30 minutes of incubation with agitation at room temperature, streptavidin conjugated to the fluorescent protein phycoerythrin was added and the plate was once again incubated on the plate shaker at room temperature for 30 minutes. Thereafter, the plate was washed to remove the unbound streptavidin-phycoerythrin, reading buffer was added to all wells and the beads were resuspended on a plate shaker for 5 minutes at room temperature. The beads (minimum of 100 beads per cytokine) were analyzed in the Luminex® 200^TM^ instrument (Invitrogen), which monitored the spectral properties of the beads while simultaneously measuring the amount of fluorescence associated with phycoerythrin. Raw data (median fluorescent intensity) was analyzed using a 5-parameter logistic method to determine the concentrations of the analytes (IL-1β, IL-6, IL-10 and TNF-α) in each sample (Luminex Xponent software 3.1).

### Data analysis

Statistical analysis were conducted using the Statistical Package for the Social Sciences (SPSS) version 23.0. Quantitative variables were reported as mean and standard deviation or as median and interquartile range, depending on data distribution. All variables were tested for normality of distribution using the Shapiro-Wilk test. Categorical variables were described as absolute and relative frequencies. To compare means between groups, the Student *t* -test was conducted. When asymmetry was observed, the Mann-Whitney test was applied. Pearson’s chi-square or Fisher’s exact tests were utilized to compare proportions between groups. To control for confounding factors, analysis of covariance (ANCOVA) was conducted. For variables with asymmetric distribution, such as cytokine levels, a logarithmic transformation of data was applied to perform the parametric test. Variable entry criteria for the multivariate model was a p-value < 0.10 in the bivariate analysis. Significance level was set at p < 0.05.

### Ethical approval

The present study was conducted at the PROTIG-HCPA outpatient clinic between May and December 2018. HCPA’s ethics committee approved the present study (protocol no. 2018/0544). Written informed consent was obtained from all participants.

## Results

### Clinical characteristics

A total of 34 non-transsexual men and 31 transsexual women were included in the study. Clinical and sociodemographic characteristics of both groups are shown in Table 1 . As shown in Table 1 , groups differed in age, religiosity, body mass index, hemoglobin and leukocyte count. The control group was older and had a higher body mass index than transsexual women. All transsexual women were on hormone therapy. Medication use in addition to hormonal therapy included reports of sporadic use of analgesics, antibiotics and anti-hypertensive drugs within the prior six months.

**Table 1:** Sociodemographic and clinical characteristics of the sample

Characteristics	Transsexual women (n = 31)		Non-transsexual men (n = 34)		p
	Mean (SD)	95%CI	Mean (SD)	95%CI	
Age^a^	29.03 (8.12)	(25.75-31.91)	37.82 (14.35)	(32.72-42.90)	**0.004**
	**Percent**	**95%CI**	**Percent**	**95%CI**	**p**
Racial background					0.416
White	56.7	(39.2-72.6)	70.6	(53.8-83.2)	
Non-white	43.3	(27.4-60.8)	29.4	(16.8-46.2)	
Socioeconomic status					
A	92.6	(76.6-97.9)	100	(82.2-100)	
B1	7.4	(2.1-23.4)	0	(0-18.8)	
Education					
High school or more	88.5	(71.0-96.0)	75.7	(58.9-87.1)	0.214
Middle school or less	11.5	(4.0-29.0)	24.3	(12.9-41.1)	
Religiosity^b^					**0.001**
Yes	46.7	(30.2-63.9)	84.8	(69.1-93.4)	
No	53.3	(26.1-69.8)	15.2	(6.6-30.9)	
General health					
Smoking					0.405
Yes	3.2	(0.6-16.2)	9.1	(3.1-23.6)	
No	96.8	(83.8-99.4)	90.9	(76.4-96.9)	
Regular physical activity					
Yes	48.4	(32.0-65.1)	57.6	(40.8-72.8)	0.462
No	51.6	(44.9-68.0)	42.4	(28.9-60.6)	
Medications (besides hormonal therapy)					0.514
Yes	48.4	(32.0-65.1)	57.9	(36.3-76.9)	
No	51.6	(44.9-68.0)	42.1	(23.1-63.7)	
	**Mean (SD)**	**95%CI**	**Mean (SD)**	**95%CI**	**p**
Body mass index	25.31 (2.96)	(24.21-26.42)	26.94 (3.48)	(25.68-28.21)	0.054
Hemoglobin^c^	14.52 (1.03)	(14.14-14.89)	15.42 (0.95)	(15.07-15.74)	**0.001**
Platelets	252,322 (44,310)	(236,069-268,575)	237,757 (40,618)	(223,354-252,159)	0.175
Leukocytes^d^	8,300 (1,773)	(7,649-8,950)	6,990 (1,469)	(6,477-7,502)	**0.002**

95%CI = 95% confidence interval; SD = standard deviation.

^a^ t = 3.005; ^b^ χ^2^ = 10.309, df = 1; ^c^ t = 3.598; ^d^ t = -3.251.

**Figure 1:**
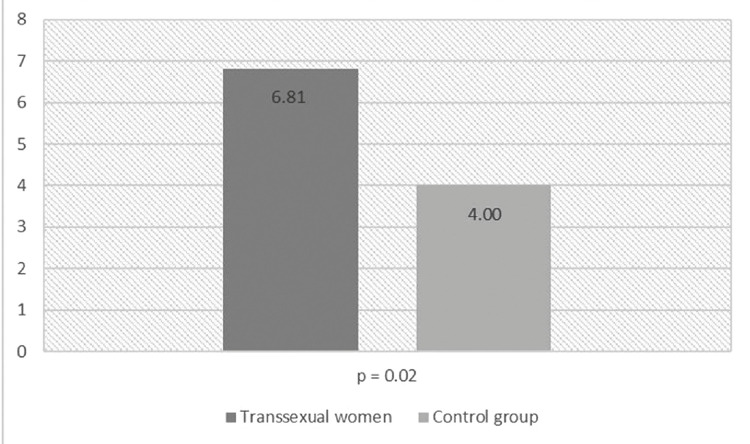
Comparison of scores obtained for the Brazilian Explicit Discrimination Scale (BEDS) between transsexual women and non-transsexual men.

**Figure 2:**
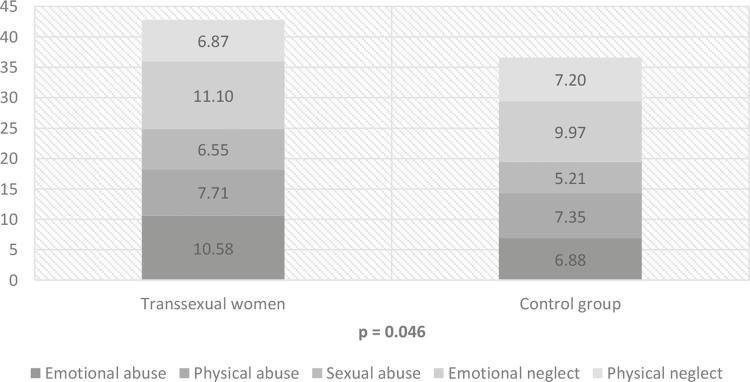
Comparison of scores obtained for the Childhood Trauma Questionnaire (CTQ) between transsexual women and non-transsexual men.

Table 2 shows measures of childhood maltreatment as well as general discrimination. Transsexual women were more exposed to prejudice and discrimination (mean = 6.81, standard deviation [SD] = 3.57) than were non-transsexual men of the control group (mean = 4.00, SD = 3.23; p = 0.002) ( Figure 1 ). Likewise, people diagnosed with GD experienced significantly more childhood maltreatment (mean = 43.03, SD = 14.04) than non-transsexual persons (mean = 36.61, SD = 11.13; p = 0.046), especially emotional and sexual abuse (respectively, p = 0.001 and p = 0.040), but not physical abuse or emotional and physical neglect. These results are illustrated in Figure 2 . People diagnosed with GD and non-transsexual men did not differ in symptoms of depression, anxiety or stress ( Table 3 ). However, transsexual women reported higher rates of suicidal ideation (p < 0.001) and previous suicide attempt (p = 0.001) than non-transsexual men.

**Table 2:** Scores obtained for the Childhood Trauma Questionnaire (CTQ) and the Brazilian Explicit Discrimination Scale (BEDS) in transsexual women vs. non-transsexual men

Traumatic events	Transsexual women (n = 31)		Non-transsexual men (n = 34)		p
	Mean (SD)	95%CI	Mean (SD)	95%CI	
Brazilian Explicit Discrimination Scale^a^	6.81 (3.57)	(5.50-8.12)	4.00 (3.23)	(2.85-5.14)	**0.002**
Childhood Trauma Questionnaire					
Emotional abuse^b^	10.58 (5.31)	(8.63-12.52)	6.88 (2.13)	(6.13-7.62)	**0.001**
Physical abuse	7,71 (4.06)	(6.27-9.24)	7.35 (3.00)	(6.30-8.40)	0.687
Sexual abuse^c^	6.54 (3.39)	(5.29-7.78)	5.20 (0.84)	(4.90-5.49)	**0.040**
Emotional neglect	11.09 (5.31)	(9.14-13.03)	9.97 (5.85)	(7.92-12.01)	0.421
Physical neglect	6.86 (2.45)	(5.94-7.71)	7.20 (2.85)	(6.20-8.19)	0.613
Total score^d^	42.81 (14.04)	(37.66-47.95)	36.61 (11.13)	(32-73-40.49)	**0.046**

95%CI = 95% confidence interval; SD = standard deviation.

^a^ t = 3.300; ^b^ t = -3.618; ^c^ t = -2.142; ^d^ t = -2.036.

**Table 3:** Measures of mood and anxiety symptoms, suicidal ideation and suicide attempt in transsexual women vs. non-transsexual men

	Transsexual women (n = 31)		Non-transsexual men (n = 34)		p
	Mean (SD)	95%CI	Mean (SD)	95%CI	
DASS-21					
Depression	3.71 (4.74)	(1.97-5.45)	2.44 (4.07)	(1.02-3.86)	0.250
Anxiety	3.19 (4.56)	(1.52-4.87)	2.15 (2.97)	(1.11-3.18)	0.273
Stress	5.84 (4.95)	(4.02-7.65)	4.15 (4.96)	(2.42-5.88)	0.174
	**Percent**	**95%CI**	**Percent**	**95%CI**	**p**
Suicidal behavior					
Ideation^a^					**< 0.001**
Yes	58.1	(39.1-75.5)	20.6	(8.7-37.9)	
No	41.9	(24.6-60.9)	79,4	(62.1-91.3)	
Attempts^b^					**0.001**
Yes	33.3	(19.2-51.2)	0	(0-10.1)	
No	66.7	(48.8-80.8)	100	(89.8-100)	

95%CI = 95% confidence interval; DASS-21 = Depression, Anxiety and Stress Scale; SD = standard deviation.

^a^ χ^2^ = 17.166, df = 2; ^b^ χ^2^ = 13.075, df = 2.

No differences were found in the levels of any cytokine between the groups ( Table 4 ). This finding did not change even after controlling for possible confounders using ANCOVA, i.e., age, religiosity, body mass index, childhood maltreatment, discrimination, leukocytes and hemoglobin count.

**Table 4:** Measures of inflammatory cytokines in transsexual women vs. non-transsexual men

	Transsexual women (n = 31)	Non-transsexual men (n = 34)	p	adjusted p*
**Median (IQR)**	**Median (IQR)**
IL-1β	0.462 (0.190-0.910)	0.304 (0.145-1.224)	0.684	0.325
IL-6	1.044 (0.116-1.445)	0.811 (0.038-2.254)	0.968	0.377
IL-10	0.312 (0.241-0.452)	0.241 (0.195-0.539)	0.474	0.552
TNF-α	2.915 (1.266-6.122)	2.422 (1.063-10.091)	0.948	0.151

IQR = interquartile range; TNF-α = tumor necrosis factor-alpha.

*Adjusted using analysis of covariance for age, religiosity, body index mass, hemoglobin, leukocytes, Childhood Trauma Questionnaire total score and Brazilian Explicit Discrimination Scale.

## Discussion

To our knowledge, this is the first study to compare both measures of childhood trauma and general discrimination between GD and non-GD populations. Additionally, no previous studies have assessed inflammatory cytokines in GD. Our main results suggest that individuals with GD experience psychological stress from childhood to adult life. However, such affliction does not seem to lead to alterations in inflammatory cytokine levels between GD and non-GD groups.

We found that transsexual women are more exposed to childhood maltreatment, especially emotional and sexual abuse, than are non-transsexual men. A systematic review showed that sexual minority populations are at a higher risk of childhood maltreatment, which is linked to psychiatric symptoms and substance abuse in adulthood.^80^ Studies have reported increased rates of sexual abuse (> 50%) in individuals with GD.^81 , 82^ Additionally, a recent metanalysis revealed that physical and sexual abuse is linked with increased risk for depression and anxiety in adulthood.^83^ In this regard, our group has found that a quarter of individuals with GD experienced some degree of childhood maltreatment,^61^ and it was associated with psychiatric disorder, suicide risk and worse psychosocial outcomes, such as sex work and unemployment, in adult life. In contrast, Bandini et al. found that childhood maltreatment in transsexual women was not only linked to psychiatric comorbidity, but also to body dissatisfaction.^5^

Even though the terminology in both diagnostic classifications has changed in the last years, in order to reduce the stigma and prejudice experienced by persons with GD while providing an adequate health care access,^84^ this population still faces this kind of marginalization within society, as seen in our results. Although our GD sample did not present psychiatric comorbidities or increased levels of mood and anxiety symptoms, it is of concern that being under such chronic stress can lead these persons to suffer from a mental disorder in the future. A higher exposure to traumatic events could also be due to the difficulties that some individuals have in protecting themselves from such stressful experiences. In addition to the lack of social and family support,^85^ this scenario puts the GD population at a high risk of developing any kind of mental disorders – such disorders, without considering the societal marginalization *per se* , are also risk factors for worse psychosocial outcomes.^86^ Moreover, our study replicates the results of a high prevalence of suicidal ideation and suicide attempts among individuals with GD, as already demonstrated.^87^ Our results revealed that suicidal ideation is highly prevalent in this population, even when psychiatric disorders that can lead to suicidal thoughts are ruled out. These results are in accordance with previous studies, which include discrimination and verbal/physical abuse as risk factors for suicidal behavior in GD.^85 , 88 , 89^ Despite the fact that we did not find an association between inflammatory markers and suicidal thoughts, other biomarkers have already been associated with suicide attempts, such as prolactin and thyroid hormones, and they could even be included in suicide risk assessment.^90^

Even though our GD sample had experienced significantly more traumatic events than the non-transsexual control group, they did not differ from the latter group in terms of psychiatric symptoms, which can be explained by several reasons. First, persons with GD were relatively young: on average, almost 9 years younger than the control group. This difference in age could have affected either negatively our control group or positively our GD group, since older persons have a worse inflammatory profile than younger ones.^91^ Second, whereas it is common for GD populations to have difficulty accessing health services,^92^ we could speculate that our GD sample had early access to these services, which likely had a positive influence on their mental health. Third, although transsexual persons struggle with more events of prejudice and discrimination than non-transsexuals, the time of occurrence of such events was not specified, which may have led to lower levels of perceived psychological stress due to the time elapsed since the events. Moreover, according to Meyer, there is a group of individuals who are exposed to minority stress but are able to promote resilience and better-coping strategies rather than psychological distress.^15^ These arguments can also explain in part the lack of differences between the groups in regards to inflammatory cytokines.

Another important issue to consider is that, to prevent our results from being influenced by possible confounders, as a result of their known effect on cytokines, we excluded diagnoses of HIV^93 - 95^ and psychiatric disorders,^32 , 35 , 96^ as well as the use of psychotropic medications.^70 , 71^ This decision may have led us to select healthy subjects who, in spite of facing stigmatization and childhood adversities, had better mental health and resilience. However, such controlled methodology also helped us to conclude that being transsexual *per se* is not the factor that contributes to worse outcomes in psychiatric comorbidities and inflammatory biomarkers – and that could later affect mental health. With this in mind, these results support the hypothesis that stigmatization, prejudice and discrimination are factors that can lead to the high prevalence of psychiatric disorders in GD.^85 , 97^

Our study has several limitations that need to be highlighted. Unfortunately, even though our methodology helped to control for possible confounders for the analysis cytokines, it limits the possibility to generalize our results, since GD is often associated with psychiatric comorbidity^24^ and HIV.^98^ Similarly, the small sample size could have prevented us from finding a statistical difference on inflammatory biomarkers. Likewise, since our entire GD sample was taking estrogen therapy, we have to consider a possible influence of steroidal hormones on these biomarkers, since steroidal hormones have a direct effect on our immune system.^99^ For example, in women, estrogen can reduce levels of inflammatory cytokines.^100^ Conversely, low levels of testosterone in men can worsen inflammatory parameters, while its administration can also reduce levels of inflammatory cytokines.^101^ Similarly, estrogen could have also influenced the higher leukocyte counts observed in transsexual women and the lower TNF-α levels when comparing to non-transsexual men (non-statistically significant difference).^99 , 102^ In regards to another biomarker, Fuss et al. found that BDNF could be reduced by estrogen in transsexual women, although it increases BDNF in non-transsexual women.^57^ This allows us to speculate that both increased levels of estrogen and reduced levels of testosterone in transsexual women can normalize BDNF levels. The absence of a non-transsexual female control group is also a limitation of this study, and our findings are only informative about IL-1β, IL-6, IL-10 and TNF- levels in transsexual women compared to non-transsexual male controls.

## Conclusion

Our findings indicate that individuals with GD experience traumatic events from childhood to adulthood when compared to non-transsexual individuals. The absence of differences in inflammatory cytokines between individuals with and without GD suggests that GD alone does not lead to inflammatory imbalance. Although these traumatic events have not influenced inflammatory biomarkers and our sample did not present elevated levels of mood and anxiety symptoms, experiencing traumatic events places this population at a high risk for psychiatric disorders and worse psychosocial outcomes. This finding further demonstrates the need for clinicians to assess traumas in people with GD, so that they can better empathize and address psychological issues related to victimization, for example by reinforcing positive gender identity and protective factors against minority stress. Better policies in order to protect this population are also necessary, such as education to the general population regarding gender identity and prejudice. Social protection measures, for example a greater penalty for those who abuse (verbally and physically) individuals with GD, would be an important mechanism to reduce violence. These approaches will promote tolerance within society in regards to gender identity and expression variances, reducing rates of discrimination. Future studies relating to inflammatory biomarkers and GD are needed to improve knowledge about the role of inflammation in GD.
